# Role of laboratory services in primary health center (PHC) outpatient department performance: an Indian case study

**DOI:** 10.1017/S1463423619000537

**Published:** 2019-07-16

**Authors:** Rahi Jain, Bakul Rao

**Affiliations:** 1 PhD student, Centre for Technology Alternatives for Rural Areas (CTARA), Indian Institute of Technology Bombay (IITB), Powai, Mumbai, India; 2 Associate Professor, Centre for Technology Alternatives for Rural Areas (CTARA), Indian Institute of Technology Bombay (IITB), Powai, Mumbai, India

**Keywords:** laboratory, laboratory service, outpatient department, PHC, laboratory, PHC performance, primary health center

## Abstract

**Background:**

In resource-constrained settings, primary health centers (PHCs) are critical for universal health coverage. Laboratory service is one of its important components. While PHC and its performance are focused, its laboratory service has been neglected in developing countries like India.

**Aim:**

To determine the role of different level of PHC laboratory services on the overall PHC performance.

**Methods:**

Cross-sectional study based on 42 PHCs of Osmanabad District, Maharashtra, India was performed. The study used levels of laboratory services in PHC as independent parameter and PHC outpatient department (OPD) visits per day (≤ 80 versus > 80) as dependent parameter. The control parameters used in the study were number of medical doctors, availability of laboratory technicians (LTs) and population coverage by PHC. Field visit was done to collect data on levels of laboratory services, but secondary source was used for other parameters. The logistic regression analysis was performed in study.

**Findings:**

The study found variation in PHC population coverage (10 788–74 702) and OPD visits per day (40–182) across PHC. Strong positive association was observed between levels of laboratory services and number of OPD visits per day in PHC. PHC offering both malaria and tuberculosis in-house testing had higher odds (4.81) of getting more OPDs (≥ 80 OPD visits per day) as compared to PHC not offering in-house testing facility for malaria and tuberculosis. This association was stronger in PHCs with lower population coverage (0–75 quartile) as compared to PHCs with higher population coverage (75–100 quartile).

**Conclusion:**

Focus on laboratory services is needed to enhance the existing PHCs performance. Skill-up gradation of existing LT could help in improving the contribution of the existing laboratories in PHC functioning.

## Introduction

Primary Health Centers (PHCs) enable cost-effective, accessible and universal coverage of health to the individual and community (World Health Organization, 1991). These PHCs are responsible to provide both preventive as well as basic curative services in poor rural areas of resource-constrained developing countries like India (MoHFW, 2012) with lack of developed private health care facilities.

Good functioning of PHC plays an important role in utilization of its services by the masses (Majumdar and Upadhyay, 2004; Monteserin *et al*., 2010). Laboratory service is recommended as an important component for good functioning of PHC. Studies have been focused on laboratory service in PHC regarding its type, quality (Jain and Rao, 2015; Devane-Padalkar *et al*., 2016), functioning (Nanjunda, 2011), utilization (Zunic *et al*., 2011; Baig *et al*., 2014) and relevance in disease control (George, 2011; Rizwan *et al*., 2013; Pakhare *et al*., 2015).

Despite such focus on PHC laboratory in the literature, 35.80 % of 25 354 Indian PHCs lack a laboratory technician (LT) to run the laboratory (Ministry of Health & Family Welfare, 2017). It is an important issue because PHC laboratory is the only diagnostic facility for people living in rural areas of developing countries like India. However, it has been neglected in the PHC settings for decades (George, 2011). A study by Planning Commission on PHCs in India has used parameters like outpatient department (OPD) visits, number of institutional deliveries or program-specific indicators as parameters to measure the PHC performance (Programme Evaluation Organization, 2001). In India, field experience indicates that district health officials may evaluate the PHC performance primarily for the non-laboratory outcomes like OPD visits and Maternal and Child Health services. This suggests that policy-makers may not be adequately convinced with laboratory relevance to ensure LT in all PHCs.

One of the reasons could be lack of literature explaining association between laboratory services and PHC performance. The knowledge of this association is important, especially in resource-limited health system settings, because policy-making is influenced by PHC overall performance rather than only laboratory performance.

Accordingly, this study aims to determine the role of different level of PHC laboratory services on the overall PHC performance. The literature has used different parameters to measure PHC performance. PHC performance has been focused in two ways namely patient-side assessment and provider-side assessment. In case of patient-side assessment, customer perceptions about the PHC services are evaluated (Sathyananda *et al*., 2018). In case of provider-side assessment, World Health Organization’s recommended framework is used (World Health Organization, 2000). The parameters used could be mapped on either the functions of the facility (governance, financing, resources and services) (Anant *et al*., 2016) or the objectives of facility (responsiveness, fairness and patient health) (Sathyananda *et al*., 2018). In the current study, the focus would be to choose a parameter which is commonly used by decision-makers to assess performance of PHC.

## Method

### Study settings

The study had adopted the cross-sectional design approach. The study used PHCs in Osmanabad District, Maharashtra, India as the case study to determine the relationship between laboratory services and PHC performance. Osmanabad district was chosen as study site because the district provides a unique PHC laboratory service setting. In the current district setting, the level of laboratory service (LLS) does not influence the number of different laboratory tests available at the PHC. Consequently, the influence of difference in number of laboratory tests available at the PHC on patient utilization of PHC is assumedly controlled in the given settings. Further, permissions to conduct study within the given time frame and resources could be obtained from the administrative authorities. Detailed explanation of PHC laboratory functioning in Osmanabad district is mentioned in Supplementary material.

### Parameters for study

In the current study, LLS was measured based on the number of different type of tests performed in PHC in-house laboratory. In the district, PHC could be categorized into three types of LLS namely: (1) all basic tests done in-house (AID), (2) all basic tests done in-house except tuberculosis test (AIDet) and (3) all basic tests done in-house except tuberculosis and malaria (AIDetm).

The parameter used for measuring PHC performance was based on three criteria: (1) it should be used by district officials in decision-making, (2) ease of availability of data across country and (3) parameter should not represent single program or disease. These criteria were used to ensure replication of study in other areas. Consequently, the parameter used to measure the overall PHC performance was OPD visits per day. The idea of measuring patient visits to PHC has been used in the literature like measuring number of patient visits for first contact (Sathyananda *et al*., 2018) and child delivery (Kashyap, 2016).

The PHC data on outcome parameter, ‘number of OPD visits’, were categorized into ‘low OPD visits/day’ PHC and ‘high OPD visits/day’ PHC. ‘Low OPD visits/day’ PHC category was for those PHCs that got less than or equal to 80 OPD visits per day. ‘High OPD visits/day’ PHC category was for those PHCs that got more than 80 OPD visits per day. Threshold for number of OPD visits per day was selected to be 80 because this is the recommended number of OPD visits for PHCs by Indian Public Health Standards. Table 1 shows the various control parameters used in the study.

**Table 1.** Control variables used in the study

Control variable	Explanation	Code	Variable type	Data source
Number of medical doctors in the PHC	This variable determines the NMD that are posted in the PHC. The PHC can have either one of three possibilities, that is, zero, one or two medical doctors.	NMD	Ordinal	Field visit to PHC
Laboratory technician availability	This variable determines the LT posted in the PHC. The PHC is assigned value of 0 or 1 based on absence or presence of LT.	LTA	Binary	Field visit to PHC
Population covered	This variable determines the population covered under each PHC. The PHC population coverage data are categorized into four intervals of 0–25 quartile (1), 25–50 quartile (2), 50–75 quartile (3) and 75–100 quartile (4).	PC	Interval	Data from DHO and Census 2011

PHC = primary health center; DHO = District Health Office.

The study also used another additional parameter, ‘number of samples collected for testing’, to determine if LLS in PHC and ‘number of samples collected for testing’ have any association. Two indicators were used for parameter ‘number of samples collected for testing’ namely: (1) ‘number of malaria samples collected’ and (2) ‘number of tuberculosis samples collected’. These indicators are transformed into binary categorical parameter type. The steps involved in creating binary parameter for indicator, ‘number of malaria samples collected’, were as follows:

1. Mean number of malaria samples collected across the PHCs (*n* = 42) in district was calculated.
2. If number of samples collected by any PHC was more than the mean value calculated in previous step, the PHC was assigned value ‘one’, else it was assigned value ‘zero’.
3. Step 2 is repeated for all the 42 PHCs.

Similar approach was used to assign categorical values for indicator, ‘number of tuberculosis samples collected’, to all PHCs (*n* = 42) collecting tuberculosis samples.

### Data collection

The data regarding PHC performance, that is, OPD per day were provided by district health office of Osmanabad from their database. The data on ‘number of malaria samples collected’ and ‘number of tuberculosis samples collected’ were collected from district health office, Osmanabad. The secondary data were from April,2015–March,2016.

The data on the ‘population covered under each PHC’ (PC) were obtained using secondary sources. The information regarding number and name of villages under each PHC was obtained from district health office of Osmanabad. The population of each village was obtained from Census 2011 database (Office of Registrar General and Census Commissioner, 2011). Consequently, population of all villages under each PHC was summated to estimate the population covered by the PHC. The calculated population coverage under each PHC was then divided into four quartiles.

Field visit to all PHCs in district (*n* = 42) was done during July–August 2015 in order to collect data on parameters namely LLS in PHC, number of medical doctors (NMD) and laboratory technician availability (LTA). During the visit, respondents were asked to provide responses for the following multiple-choice objective questions to collect the data:

1. What type of tests was performed in the PHC in-house laboratory? ‘All basic tests’, ’All basic tests but not tuberculosis test’, ‘All basic tests but not malaria and tuberculosis test’.
2. How many medical doctor/s are posted to PHC? Zero, one and two.
3. Does the PHC have LT posted? Yes or No.

The oral informed consent was obtained from each respondent regarding the purpose of questions and use of the responses. The respondents were explained the meaning of question both in English and in local language namely Hindi. The respondent preferred for providing responses was medical doctor or LT posted at PHC. However, in one PHC, no medical doctor or LT was available during visit so response from other PHC staff was obtained. The field data regarding each PHC were shown to district health officials to ensure validity of responses because these officials know PHC staff details and provide laboratory test consumables to PHC.

### Data analysis

The logistic regression analysis was performed to estimate the effect of the main parameter as well as control parameters on dependent parameter for determining significant predictors of the PHC performance. The PHC performance parameter, ‘number of OPD visits’, was used as the dependent parameter, *y*. The dependent parameter categories namely ‘low OPD visits/day’ PHC and ‘high OPD visits/day’ PHC were binary coded as *y* = 0 and *y* = 1, respectively. The analysis was done in R software. The study performed univariate and multivariate logistic regression in two scenarios.

In scenario one, univariate logistic regression was performed to estimate the influence of LLS, NMD, LTA and PC on the odds of PHC performance as ‘high OPD visits/day’ state (*y* = 1), rather than in ‘low OPD visits/day’ state (*y* = 0). Multivariate logistic regression was performed to estimate the combined influence of each of the main and control parameter on the odds of PHC performance as ‘high OPD visits/day’ state (*y* = 1), rather than in ‘low OPD visits/day’ state (*y* = 0).

In scenario two, both univariate and multivariate logistic regression were performed using the approach adopted in scenario one, except that fourth quartile of PC parameter, that is, 75–100 quartile, was omitted from the analysis. The scenario two was performed because the results from scenario one (for details see ‘Results’ section) suggested that PHC over 75 quartile of population was also having influence on OPD visits/day in the PHC.

In another analysis, the conditional probability of number of samples collected for malaria and tuberculosis test with different LLS in PHC was determined. Cramer’s *V* test for significance test was performed.

## Results

### Characteristics of the PHC

The characteristics of the parameters are shown in Table 2. In this study, daily OPD lies in range of 40–182. The number of PHCs is equally divided between daily OPD visits of less than 80 and daily OPD visits of more than 80. In terms of human resources, 20 PHCs have two medical doctors posted while remaining PHCs have only one medical doctor. Although, none of the PHCs is without medical doctor, many PHCs (45.24 %) do not have the LT, so they cannot perform the malaria and tuberculosis tests. All the PHCs with LT (*n* = 23) provide malaria blood smear examination for malarial parasite. Among the 23 PHCs with LT, 9 PHCs have been given training for tuberculosis diagnosis, so they in addition to other tests, also perform sputum testing for tuberculosis diagnosis. In terms of population covered by the PHC, the average population coverage per PHC is 32 317. However, individual PHC population coverage can vary from 10 788 to 74 702.

**Table 2.** Descriptive statistics of variables

Variable	*n* (# of PHCs)	%	Range	Mean	SD	Median
OPD/day	42	100.00	40–182	82	26.06	80
Low (< = 80)	21	50.00	40–79	64	11.39	67
High (> 80)	21	50.00	82–182	100	23.50	92
*LLS*	42	100.00				
AIDetm	19	45.24				
AIDet	14	33.33				
AID	9	21.43				
*NMD*	42	100.00				
0^a^	0	0.00				
1	22	52.38				
2	20	47.62				
*LTA*	42	100.00				
0	19	45.24				
1	23	54.76				
*PC*	42	100.00	10 788–74 702	32 317	12 848.49	29 120
0–25	11	26.19	10 788–25 066	19 378	3805.40	19 819
25–50	10	23.81	25 165–28 994	27 212	1470.95	27 524
50–75	10	23.81	29 246–37 007	32 498	2451.91	32 270
75–100	11	26.19	40 138–74 702	49 732	10 541.46	44 975

a This is not further used in the study, as it is not present for any PHC.

PHC = Primary Health Center; OPD = Outpatient Department; LLS = Level of laboratory services; AIDetm = all basic tests done in-house except tuberculosis and malaria; AIDet = all basic tests done in-house except tuberculosis test; AID = all basic tests done in-house; NMD = number of medical doctors; LTA = laboratory technician availability; PC = population covered under each PHC.

### Role of LLS in overall PHC performance

As Table 3 shows, the logistic regression for ‘low OPD/day’ and ‘high OPD/day’ identifies PHC with LLS having ‘AID’ to be significant. This means that probability (more precisely, odds) of PHC having AID satisfies high OPD (*y* = 1) is 4.81 times higher than probability of AIDetm satisfies the same. The ratio (4.71) to be significantly distinct from 1 indicates that it is statistically significant predictor of being in ‘high OPD visits/day’ state, rather than in ‘low OPD visits/day’ state. Among control parameters, PC having ‘75–100 quartile’ is significantly influencing the PHC performance. Even, the multivariate logistic regression has obtained the same results.

**Table 3.** Logistic regression analysis result to estimate the role of LLS in PHC performance

Independent variable	Univariate analysis		Multivariate analysis	
	Low OPD/day (*y* = 0) versus High OPD/day (*y* = 1)			
	Crude odds ratio	90 % CI	Adjusted odds ratio	90 % CI
LLS (ref = AIDetm)				
AIDet	1.03	0.31–3.35	0.95	0.26–3.35
AID	4.81*	1.15–26.31	4.21	0.82–27.11
NMD (ref = One MD)				
Two MD	2.17	0.78–6.23	2.03	0.61–7.1
LTA^a^ (ref = No)				
Yes	1.79	0.64–5.1		
PC (ref = 0–25 quartile)				
25–50 quartile	1.75	0.41–7.92	1.12	0.21–5.87
50–75 quartile	1.17	0.26–5.26	1.11	0.22–5.53
75–100 quartile	4.67*	1.08–23.34	5.34*	1.13–30.27

Regression analysis after removing 75–100 percentile PHC population coverage data				
Independent variable	Low OPD/day (*y* = 0) versus high OPD/day (*y* = 1)			
	Crude odds ratio	90 % CI	Adjusted odds ratio	90 % CI
LLS (ref = AIDetm)				
AIDet	1.67	0.39–7.24	1.61	0.37–7.14
AID	6.25*	1.26–39.25	5.52	0.9–43.16
NMD (ref = One MD)				
Two MD	2.00	0.6–7.03	1.23	0.29–4.93
LTA^a^ (ref = No)				
Yes	2.81	0.83–10.49		
PC (ref = 0–25 quartile)				
25–50 quartile	1.75	0.41–7.92	1.08	0.20–5.56
50–75 quartile	1.17	0.26–5.26	1.03	0.20–5.12

a LTA is not considered for multivariate logistic regression because LLS is dependent on LTA.

* *P* < 0.1.

LLS = Level of laboratory services; PHC = Primary Health Center; OPD = Outpatient Department; CI = confidence interval; AIDetm = all basic tests done in-house except tuberculosis and malaria; AIDet = all basic tests done in-house except tuberculosis test; AID = all basic tests done in-house; NMD = number of medical doctors; LTA = laboratory technician availability; PC = population covered under each PHC.

During the logistic regression, PHCs with very high population coverage, that is, 11 out of 42 PHCs that are present in ‘75–100 quartile’ category of PC parameter, were removed from the study (Table 3). The univariate logistic regression for the ‘low OPD visits/day’ and ‘high OPD visits/day’ identifies PHC with LLS having ‘AID’ to be significant, but no control parameter is found significant. In case of multivariate logistic regression, none of the parameters are found significant. Further, the odds ratio for LLS is higher when PHCs in ‘75–100 quartile’ category of PC parameter is not considered in regression compared to regression done using 42 PHCs.

### Association between LLS and number of laboratory samples collected at the PHC

The conditional probability of number of samples collected for malaria and tuberculosis test with different LLS in PHC is determined (Table 4). The study finds that in case of malaria sample collection, the probability of above-average sample collection is more in case of PHC with LLS having ‘AID’ (0.78) as compared to PHC with LLS having ‘AIDet’ (0.36) or ‘AIDetm’ (0.47). Similarly, the probability of above-average tuberculosis sample collection is more in case of PHC with LLS having ‘AID’ (0.89) and ‘AIDet’ (0.57) as compared to ‘AIDetm’ (0.26). The medium strength of association is observed between collection of samples for test and LLS using Cramer’s *V* test for both the malaria (0.49) and tuberculosis (0.31) sample collection.

**Table 4.** Conditional probability of number of samples collected for testing with different LLS

Condition (*n*)	No. of malaria samples collected		No. of tuberculosis samples collected	
LLS	≤ Mean (21)	> Mean (21)	≤ Mean (21)	> Mean (21)
No condition	0.50	0.50	0.50	0.50
AIDetm (19)	0.53	0.47	0.74	0.26
AIDet (14)	0.64	0.36	0.43	0.57
AID (9)	0.22	0.78	0.11	0.89
	Cramer’s *V* = 0.49, df = 1		Cramer’s *V* = 0.31, df = 1	

LLS = Level of laboratory services; AIDetm = all basic tests done in-house except tuberculosis and malaria; AIDet = all basic tests done in-house except tuberculosis test; AID = all basic tests done in-house.

## Discussion

PHC is an important health care facility in rural areas, but the approach to ignore laboratory in PHC facility may not be appropriate to maximize the PHC performance. The study showed that association between LLS in PHC and number of OPD visits per day is positive. This positive association during univariate logistic regression analysis is found significant at 90% confidence interval for PHC providing all tests, that is, PHC with AID. The very high odds ratio (4.81, 4.21, 6.25 and 5.52) suggests the strength of this association. In the literature, the strong positive correlation was obtained between laboratory service-related parameters and overall hospital performance (composite of patient results, staff and work system result, hospital efficiency and effectiveness result and flexibility performance) for Jordanian Hospitals (Ali and Alolayyan, 2013). The study on US hospitals showed that clinical technology inclusive of laboratory technology drives the hospital clinical quality and financial performance (Li and Collier, 2000).

However, the LLS in PHC was not found to be a significant predictor of overall PHC performance in multivariate analysis, which was unexpected. Further, the large confidence interval indicates that some precautions are needed in interpreting the absolute effect of LLS in PHC on PHC performance. These findings suggest that LLS in PHC could be a strong trigger to improve the PHC performance, but alone it is not an enough condition to improve the PHC performance. In the Indian context, patient could access public laboratory facility only on referral from medical doctor (Jain and Rao, 2015). Thus, the laboratory can help the physician in better decision-making, which could lead to better PHC performance. The literature had suggested that laboratory results could contribute up to two-third of medical decision-making (Forsman, 1996). Further, the literature had identified various reasons that could disrupt physician role in PHC like lack of resources (Hazra and Das, 2016) and medical doctor motivation (Shah *et al*., 2016).

The study showed that LLS is more relevant for PHCs with population catchment area less than or equal to IPHS recommendations. Odds ratio of LLS in PHC is higher when PHC with very high population coverage is not considered vis-à-vis when considered. The PHCs with high population coverage can get more OPDs because of their catchment area. However, PHCs with smaller population coverage may need to provide better services so that patients with different health care needs willingly visit the PHC. In West Bengal, India, a strong correlation was reported between number of OPD hours and patient perception of PHC service quality (Bhattacharya, 2015). In another case study, it was reported that inadequate PHC service was affecting the number of patients whose needs could be catered by the PHC (Hazra and Das, 2016). The reduction in antenatal checkups from first to fourth checkup was observed due to poor PHC facilities (Dehury and Samal, 2016).

The study showed that the level of training provided to LT influences PHC performance. The PHC performance shows very low and insignificant positive association with LT trained only in performing malaria test as compared to LT trained in both malaria and tuberculosis test. This suggests that Osmanabad district can enhance the overall performance of its 14 PHCs by training LT to conduct tuberculosis testing. This could allow better utilization of available resources. Based on the literature, an average annual cost of delivering health care services at an Indian PHC could be around $113 683–158 883 (1USD=64.69INR) (Prinja *et al*., 2016). This is relevant for resource-constrained countries like India that need to maximize the cost-effectiveness of its health care facilities.

The study showed that the PHCs with better LLS have reported more sample collection for testing. This suggests that higher LLS in PHC could increase the laboratory service utilization, which can help in improving PHC services. In the literature, a study had reported that change in LLS like reduction in laboratory turnaround time significantly reduced the patient length of stay (Holland, Smith and Blick, 2018).

The district used for the current study has daily OPD visits range (40–182) similar to the OPD visits range (25–150 OPD visits/day) reported in the literature (Rizwan *et al*., 2013; Dar, 2015; Raut-Marathe *et al*., 2015). In terms of population covered by the PHC, the average population coverage per PHC (32 317) is similar to national average population coverage of around 32 854.77 per PHC (Office of Registrar General and Census Commissioner, 2011; Ministry of Health & Family Welfare, 2017). The variation in population coverage across PHCs (10 788–74 702) is similar to population coverage range of 7000–57 918 across PHCs reported in the literature (Bhatt and Joshi, 2013; Rizwan *et al*., 2013; Prinja *et al*., 2016; Tushi and Kaur, 2017). This reflects that the overall context in which PHC functions in the current study district is not far away from overall national scenario. Hence, relevance of this study results could extend beyond the district to whole country.

Finally, this study is relevant as it strengthens the need to put more focus on public laboratory service in developing countries like India. It provides an evidence to decision-makers that laboratory is important in enhancing PHC performance and achieving the greater goal of universal health coverage. The study has an important policy implication. It suggests that mere availability of laboratory tests may not be an enough criterion to make patients visit the PHC. It may be important to have better LLS like performing in-house tests.

In-house tests may help in reducing the turnaround time for the tests which could help doctors to quickly diagnose and provide appropriate medical services. The in-house laboratory testing is more relevant in primary health care settings, which is providing basic laboratory tests like microscopy-based tuberculosis test and malaria test. These tests take only few minutes to provide results, but in rural field settings of developing country like India, the delay in results could be as high as few days due to an issue in accessing laboratory facilities. For example, during field survey, interactions with locals and PHC staff suggested that many PHCs are not connected with any form of public transport to other nearby laboratory facility. Further, even if, public transport facility is available, the transport facility is limited to one or two trips per day. Additionally, the PHC commonly sends a staff to other laboratory facility only once a day.

One of the limitations of this study is small sample size restricted to a limited geographic area. This could be one reason for lower statistical power. Further, the study is not performed temporally but only longitudinally. Another possible weakness is that this study could have missed other laboratory-related important parameters that may play an important role in PHC performance. Additionally, the study does not consider the role of PHC staff other than LT and medical doctor in influencing the relationship between LLS in PHC and PHC performance.

## Conclusion

The study concludes that laboratory services could play an important role in maximizing the PHC performance. Higher LLS in PHC could help in getting more visits in the OPD. The training of existing LTs could be a cost-effective approach in resource-constrained settings to maximize the returns from the existing medical workforce in PHCs. Finally, study found that PHCs with lower population coverage could benefit from higher LLS as compared to other PHCs in enhancing their performance in terms of number of OPD visits per day.

## Supplementary material

For supplementary material accompanying this paper visit https://doi.org/10.1017/S1463423619000537. click here to view supplementary material
