# Supplementary material for: Role of laboratory services in primary health center (PHC) outpatient department performance: an Indian case study
Source: Prim Health Care Res Dev. 2019 Jul 16;20:e112. doi: 10.1017/S1463423619000537 (PMC6635801; doi:10.1017/S1463423619000537)
Supplement: Supplementary file 1 [file S1463423619000537sup001.doc]

**PHC laboratory services in PHCs of Osmanabad District: Study Area**

Indian public healthcare system is a three-tier system that consists of tertiary, secondary and primary healthcare level. Both tertiary and secondary healthcare level facilities are present in only urban areas, whereas primary healthcare level facilities are present in both urban and rural areas (Planning Commission of India, 2008).

The Osmanabad district lies in Maharashtra State, India and is subdivided into eight blocks. The district public healthcare system has both 12 secondary healthcare facilities (SHF) and 248 primary healthcare facilities (PHF). The SHF consists of district hospital (one), maternity and child hospital (one), sub-district hospital (three) and community health center (seven), whereas PHF consists of PHC (42) and subcenter (206). SHF facilities are present in urban areas and serve the complete district population of 1.7 million out of which 84% live in rural areas (Office of Registrar General and Census Commissioner, 2011). Primary healthcare facilities are present only in rural areas. PHCs are the *first point of contact* for the rural population to access medical doctor, laboratory technician and laboratory facilities (MoHFW, 2012). This makes good functioning of PHC critical for rural populace healthcare.

According to Indian Public Health Standard (IPHS) 2012, all PHCs should have two medical doctor (MD) and should cover the population of maximum 30,000 to ensure quality and standardized laboratory services across the nation (MoHFW, 2012). In terms of laboratory services, all PHCs should have one laboratory technician (LT) and provide 27 basic laboratory tests (Jain and Rao, 2015). Malaria blood smear examination for malarial parasite is recommended in IPHS standards and sputum testing for tuberculosis diagnosis is desirable as per the IPHS standards (MoHFW, 2012). However, the PHCs in Osmanabad perform few basic kit-based tests that are provided by the district officials rather than all 27 basic laboratory tests.

According to national policy guidelines, the PHCs who do not have particular facility are recommended to refer the patient to referral health care facility where the facility is available (MoHFW, 2005). While, Osmanabad PHCs follow the recommendations, in case of malaria and tuberculosis diagnosis a different approach is adopted (Figure 1). The PHC that do not have malaria or tuberculosis or both testing facilities collect the patient sample, process the sample and send it to a nearby public healthcare facility for testing. The result obtained from sample testing is communicated back to the PHC and from PHC to patient. Therefore, from the patient’s perspective, all the PHCs can perform basic kit based test, malaria and tuberculosis test because patient do not have to go to any different laboratory facility for diagnosis.


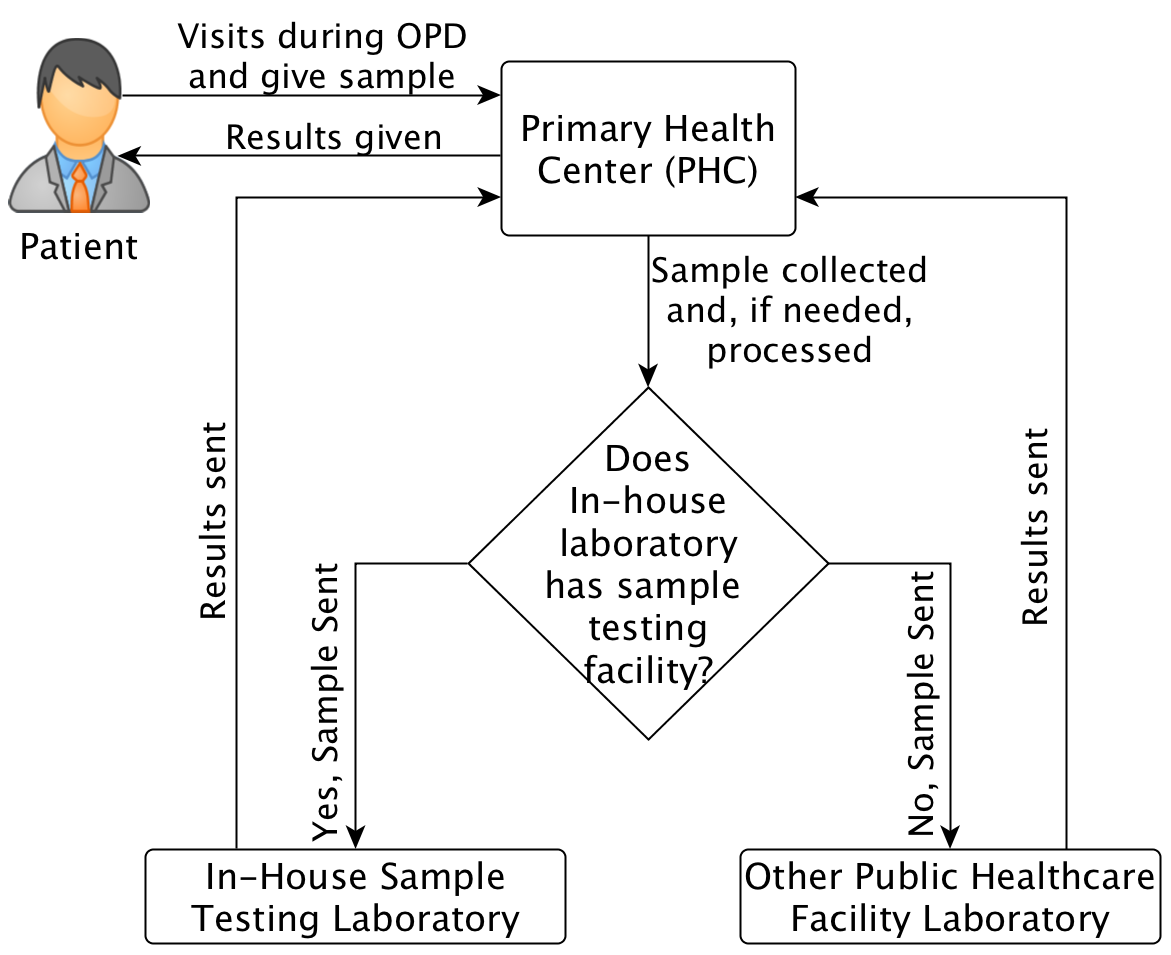


Figure 1: PHC Laboratory service in Osmanabad District

***Reference***:

Jain, R. and Rao, B. (2015) ‘Medical diagnostic laboratories provisioning of services in India’, *CHRISMED Journal of Health and Research*, 2(1), pp. 19–31.

MoHFW (2005) *National Rural Health Mission: Framework for Implementation (2005-2012)*. New Delhi, India.

MoHFW (2012) *Indian Public Health Standards (IPHS) for Primary Health Centres Revised Guidelines 2012*. New Delhi, India.

Office of Registrar General and Census Commissioner (2011) *Census 2011: Population Enumeration Data*, *Ministry of Home Affairs*. Available at: http://www.censusindia.gov.in/2011census/population_enumeration.html (Accessed: 11 January 2016).

Planning Commission of India (2008) ‘Health and Family Welfare and AYUSH’, in *Eleventh Five Year Plan (2007-2012): Social Sectors (Volume II)*. New Delhi: Oxford University Press, pp. 57–127. Available at: http://planningcommission.nic.in/plans/planrel/fiveyr/index9.html.
